# Structure of the *Cannabis sativa* olivetol‐producing enzyme reveals cyclization plasticity in type III polyketide synthases

**DOI:** 10.1111/febs.15089

**Published:** 2019-10-28

**Authors:** Lewis J. Kearsey, Nicole Prandi, Vijaykumar Karuppiah, Cunyu Yan, David Leys, Helen Toogood, Eriko Takano, Nigel S. Scrutton

**Affiliations:** ^1^ Manchester Institute of Biotechnology and School of Chemistry The University of Manchester UK; ^2^ BBSRC/EPSRC Synthetic Biology Research Centre SYNBIOCHEM The University of Manchester UK; ^3^ EPSRC/BBSRC Future Biomanufacturing Research Hub The University of Manchester UK; ^4^ Present address: Immunocore Limited Abingdon Oxfordshire OX14 4RY UK

**Keywords:** cannabinoid pathway, olivetol synthase, olivetolic acid, structure‐guided mutagenesis, type III polyketide synthase

## Abstract

In the native pathway to therapeutic cannabinoid biosynthesis in *Cannabis sativa*, the three‐step production of a key intermediate, olivetolic acid, is catalysed by the enzymes tetraketide synthase (TKS; linear tetraketide intermediate production in two stages) and olivetolic acid cyclase (OAC; final C2 → C7 aldol condensation). In the absence of OAC, a nonenzymatic C2 → C7 decarboxylative aldol condensation of the tetraketide intermediate occurs forming olivetol. TKS is a type III polyketide synthase, and the question arises why it is unable to form olivetolic acid directly, but instead forms this unwanted side product. We determined the TKS, CoA complex structure, and performed structurally guided mutagenesis studies to identify potential residues responsible for cyclization pathway discrimination in type III polyketide synthases. Prior studies suggested an ‘aldol switch’ is necessary to allow linear tetraketide intermediate release prior to cyclization, thereby enabling subsequent olivetolic acid production by OAC. However, our studies do not support the presence of a universal or predictable ‘aldol switch’ consensus sequence. Instead, we propose the mode of ordered active site water activation between type III polyketide synthases catalysing different cyclization mechanisms is subtle and homologue‐specific. Our work indicates that subtle structural variations between homologous enzymes can have a major mechanistic impact on the catalytic outcome. This highlights the importance of embedding high‐resolution structural analysis of multiple enzyme homologues with classical site‐directed mutagenesis studies when investigating highly similar enzymes with different mechanistic pathway outcomes.

**Enzymes:**

TKS, http://www.chem.qmul.ac.uk/iubmb/enzyme/EC2/3/1/206.html; OAC, http://www.chem.qmul.ac.uk/iubmb/enzyme/EC4/4/1/26.html; chalcone synthase, http://www.chem.qmul.ac.uk/iubmb/enzyme/EC2/3/1/74.html; stilbene synthase, http://www.chem.qmul.ac.uk/iubmb/enzyme/EC2/3/1/95.html; 2‐PS, http://www.chem.qmul.ac.uk/iubmb/enzyme/EC2/3/1/-.html.

**Accession numbers:**

The atomic coordinates and structure factors for the crystal structure of TKS have been deposited in the Protein Data Bank with accession number http://www.rcsb.org/pdb/search/structidSearch.do?structureId=6GW3.

## Graphical abstract

Tetraketide synthase from *Cannabis sativa* is a type III polyketide synthase involved in cannabinoid production. Unlike chalcone and stilbene synthases, it cannot catalyse classical cyclization reactions to generate chalcone or stilbene acid products. Instead, it releases a linear tetraketide product that undergoes a non‐enzymatic C2→C7 decarboxylative aldol condensation to form a stilbene. In this study by Nigel Scrutton and co‐authors, structure determination and mutagenesis studies are performed to investigate mechanistic details of this *Cannabis sativa* tetraketide synthase.

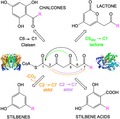

**Abbreviations**
∆^9^‐THC: tetrahydrocannabinol
2‐PS: 2‐pyrone synthase
APT: aromatic prenyltransferase
CBD: cannabidiol
CBDAS: cannabidiolic acid synthase
CBGA: cannabigerolic acid
CHS: chalcone synthase
CSD: sulfinic acid
HTAL: hexanoyl triacetic acid lactone
LC/MS: liquid chromatography mass spectrometry
OAC: olivetolic acid cyclase
OLA: olivetolic acid
PDAL: pentyl diacetic lactone
STS: stilbene synthase
TB: Terrific broth
THCAS: tetrahydrocannabinolic acid synthase
TKS: tetraketide synthase

## Introduction

Cannabinoids are a class of secondary metabolites found exclusively in the plant species *Cannabis sativa* 1. Tetrahydrocannabinol and cannabidiol (CBD) are the two main cannabinoids currently investigated for their therapeutic potential 2. They are most commonly prescribed to manage the side effects of nausea and vomiting associated with chemotherapy 3. Additional therapeutic uses are becoming more commonplace, including the treatment of anorexia in HIV patients and the reduction of spasticity in multiple sclerosis 4. In addition, olivetolic acid, the precursor of the monoaromatic cannabinoids tetrahydrocannabinol and CBD, is known to possess antimicrobial, cytotoxic and photo‐protective activities 5, 6.

**Scheme 1:**
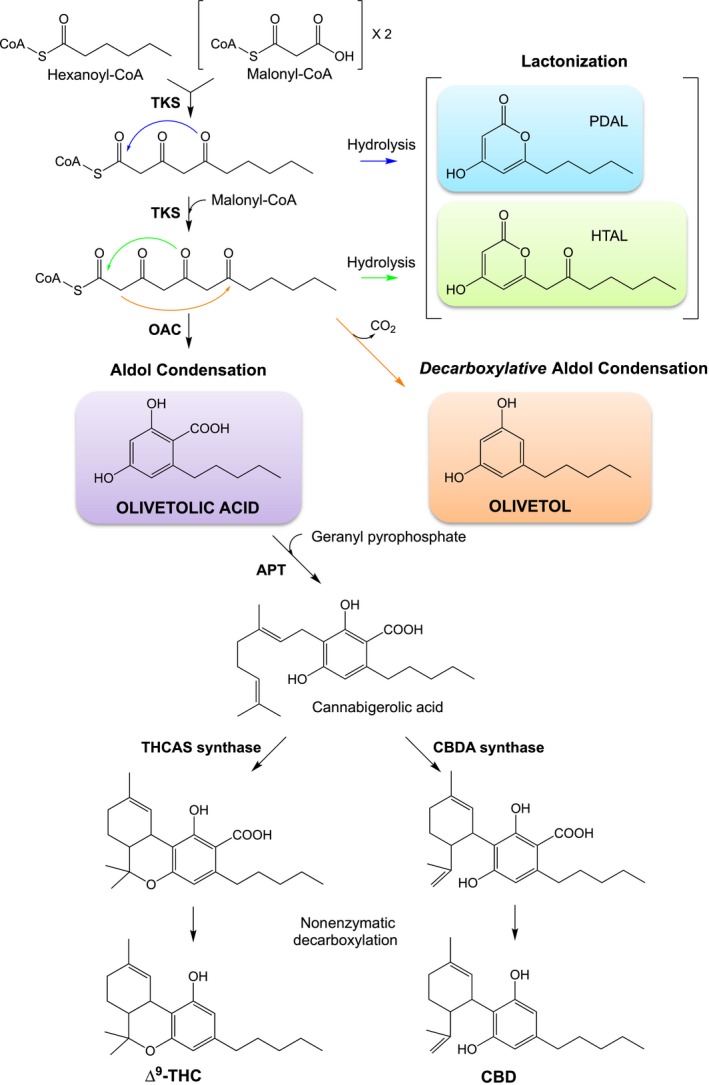
Biosynthetic pathway for the production of the two cannabinoids ∆^9^‐THC and CBD from hexanoyl‐CoA and malonyl‐CoA in *Cannabis sativa*. By‐products are HTAL and PDAL, respectively. The enzymes in the pathway are TKS, OAC, APT, THCAS and CBDAS. TKS reaction arrow colour coding of blue, green and orange corresponds to the formation of products PDAL, HTAL and olivetol, respectively.

The biosynthetic pathway for the production of tetrahydrocannabinol and CBD begins with the iterative condensation of three malonyl‐CoA and one hexanoyl‐CoA molecule to form a linear tetraketide intermediate (Scheme 1) 7, 8. This is catalysed by a tetraketide synthase (TKS), also known as olivetol synthase 8. Olivetolic acid cyclase (OAC) then performs C2 → C7 aldol condensation of the linear tetraketide intermediate to form olivetolic acid as the major product 7. Cannabigerolic acid (CBGA) is subsequently formed by the addition of geranyl pyrophosphate by aromatic prenyltransferase (APT). This is followed by a reaction with either tetrahydrocannabinolic acid synthase (THCAS) or cannabidiolic acid synthase (CBDAS), and a nonenzymatic decarboxylation to generate tetrahydrocannabinol and CBD, respectively (Scheme 1) 9.

**Scheme 2:**
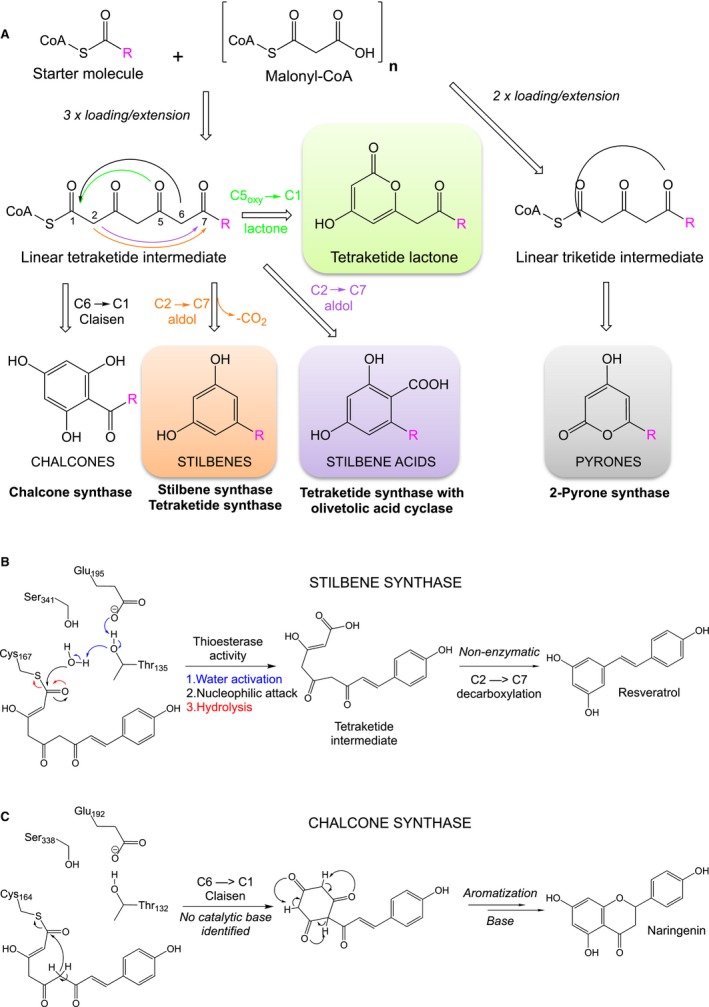
Polyketide synthase type III overview. (A) Schematic overview of the cyclization of linear tetraketide intermediates by type III polyketide synthases. (B) Proposed mechanism of the C1 thioesterase cleavage of the linear tetraketide intermediate from the active site of STS (adapted from 12). (C) Proposed mechanism of naringenin formation by CHS via C6 → C1 Claisen condensation (adapted from 14).

TKS is a member of the type III polyketide synthase family 10, with high sequence similarity to the well characterized chalcone synthase (CHS; 66%) 11 and stilbene synthase (STS; 60%) 12. These enzymes undergo similar malonyl‐CoA loading and extension steps to generate an enzyme‐bound linear tetraketide intermediate (Scheme 2), but vary in the number of condensations and the type of starter molecule. In one case, 2‐pyrone synthase (2‐PS) catalyses only two chain extending condensations to form a triketide intermediate prior to cyclization (Scheme 2A) 13. This family of enzymes also utilize different cyclization mechanisms to generate distinct chemical scaffolds. For example, with coumaroyl‐CoA as the starter molecule, CHS performs a C6 → C1 intramolecular Claisen cyclization of the tetraketide intermediate to form the chalcone, naringenin 11. The reactions of STS with the same substrates diverge after tetraketide intermediate formation, with cleavage of the C1 thioester linkage occurring between the product and the catalytic triad cysteine, known as the ‘aldol switch’. This in turn leads to a nonenzymatic C2 → C7 decarboxylative aldol condensation reaction that forms resveratrol (Scheme 2A) 12. However, for type III polyketide synthases additional C5_oxy_ → C1 lactonization reactions of the triketide and tetraketide intermediates can occur, leading to a ‘derailment’ of the normal catalytic cycle (Scheme 2A).

In the case of cannabinoid biosynthesis, cyclization of the tetraketide intermediate to olivetolic acid is a C2 → C7 aldol condensation catalysed by OAC (Scheme 2A). As no functional complex has been detected between TKS and OAC 9, this suggests the linear tetraketide intermediate must be released from TKS to interact with OAC. Therefore, TKS may possess an STS‐like mechanism of C1 thioester cleavage (Scheme 2B) 12. In the absence of OAC, olivetol is the major product presumably via a nonenzymatic STS‐like C2 → C7 decarboxylative aldol condensation of the released tetraketide intermediate (Scheme 1) 12.

Here, we have determined the crystal structure of TKS in the presence of CoA. We have also performed a structure‐guided mutagenesis study to investigate why the tetraketide intermediate is released, enabling OAC to catalyse olivetolic acid production. Comparative structural analyses between TKS, CHS and STS were performed to investigate the nature of the ‘aldol switch’ 12 in TKS. This was to gain insight into why STS‐like C2 → C7 decarboxylative aldol condensation most likely occurs with TKS instead of a CHS‐like C6 → C1 Claisen cyclization (Scheme 2C) 14. A variety of residues lining the substrate‐binding site of TKS were then targeted by mutagenesis to determine whether they impacted on the relative proportions of the products formed in the presence and absence of OAC.

## Results and Discussion

### Product profile of TKS

**Figure 1:**
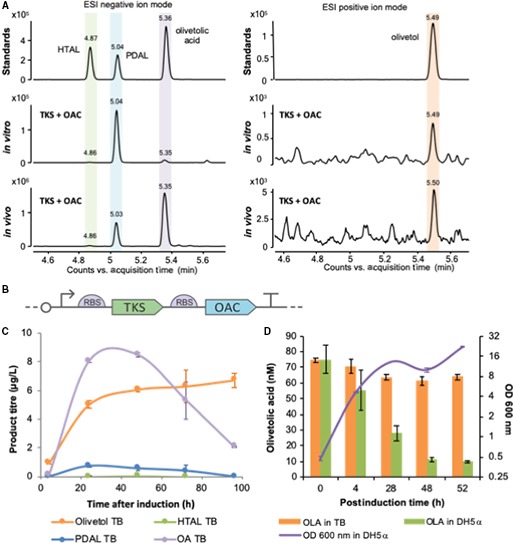
Production profiling of TKS and OAC under *in vitro* and *in vivo* conditions. (A) LC Q‐TOF MS qualitative analysis of the products obtained by TKS expressed together with OAC *in vitro* and *in vivo*. Standards: commercial chemical standards. *In vitro*: products obtained in a biotransformation assay with purified TKS and OAC supplied with substrates. *In vivo:* products obtained when expressing TKS and OAC in *Escherichia coli* as shown in (B) at 20 °C after induction in TB media. Negative controls for both conditions are shown in Fig. S1. Samples were run in ESI negative ion mode. The chromatogram shown is composed by two merged extracted ion chromatograms (*m*/*z* = 223.098 and 181.087), and the single chromatograms are shown in Fig. S1. Samples run in ESI positive ion mode, and chromatogram shown is extracted ion chromatogram (*m*/*z* = 181.12). The retention times of authentic standards are HTAL (4.8 min), PDAL (5.03 min), olivetolic acid (5.3 min) and olivetol (5.59 min). (B) Schematic figure of the expression construct. (C) Quantification of olivetolic acid and by‐products with time obtained under *in vivo* conditions. Samples from *E. coli* transformed with construct (B) were collected at different time points after induction, grown at 20 °C in TB media. The compounds were quantified by LC combined with triple quadrupole MS (LC‐MS/MS) using reference standards at known concentrations (see Material and Methods). Data points are average of two biological replicates, and bars represent standard deviation. (D) Olivetolic acid degradation in the presence of *E. coli*. Cultures of a GFP‐expressing plasmid (pBbB2c‐GFP) 16 in TB medium (5 mL; starting OD_600 nm_ of 0.1) were incubated at 37 °C until the OD_600 nm_ reached 0.8. Olivetolic acid (100 μm) was added to this culture followed by a further incubation at 30 °C for 52 h at 200 r.p.m. Control incubations of olivetolic acid were performed in TB medium only with no *E. coli* present. Periodic culture sampling was performed, and OD_600 nm_ was measured as well as olivetolic acid extracted in ethyl acetate, dried, resuspended in methanol and analysed by LC/MS. Data points are the average of three replicates, and bars represent standard deviation. OLA in TB: media only with the addition of olivetolic acid standard; OLA in DH5α: *E. coli* strain DH5α with the addition of olivetolic acid standard. OD_600 nm_ DH5a: Growth of *E. coli* strain DH5α.

The low catalytic rate of purified TKS with hexanoyl‐CoA (< 0.02 s^−1^) 8 necessitated biotransformations to be performed for 16 h, followed by product identification (Figs 1A and S1) and quantification by liquid chromatography mass spectrometry (LC‐MS). A major product generated by TKS with malonyl‐CoA and hexanoyl‐CoA was identified as olivetol (416 ± 96 nm), as seen in previous studies 8. This is likely produced via an STS‐like nonenzymatic C2 → C7 decarboxylative aldol condensation of the tetraketide intermediate after release from TKS (Scheme 2). Similar quantities of pentyl diacetic lactone (PDAL) were detected; however, the exact concentration PDAL produced was variable and dependent on the enzyme batch (Table S1). PDAL formation occurs via lactonization of the linear triketide intermediate 15 and is considered to be a premature ‘derailment’ by‐product (Scheme 1) 1. A similar reaction with the tetraketide intermediate generated trace titres of the equivalent lactone hexanoyl triacetic acid lactone (HTAL; 5.4 ± 3.7 nm). These low levels of HTAL relative to olivetol agree with prior kinetic studies that suggested that nonenzymatic decarboxylative aldol condensation proceeds with a higher efficiency than lactonization 1. As expected, no olivetolic acid was detected; however, small titres (13.7 ± 4.0 nm or 3.1 μg·L^−1^) were present in comparable *in vitro* reactions with both TKS and OAC.

We assembled a dual enzyme construct expressing both TKS and OAC under the control of a single inducible *tetR* promoter (Fig. 1B) 16. *In vivo* studies were performed to investigate the product profile of this plasmid construct in *Escherichia coli*. Interestingly, a difference was seen in the relative proportions of olivetolic acid between *in vitro* and *in vivo* reactions (Fig. 1A). The PDAL production level differs from previous *in vivo* studies in *E. coli* that detected only trace levels of PDAL.

**Figure 2:**
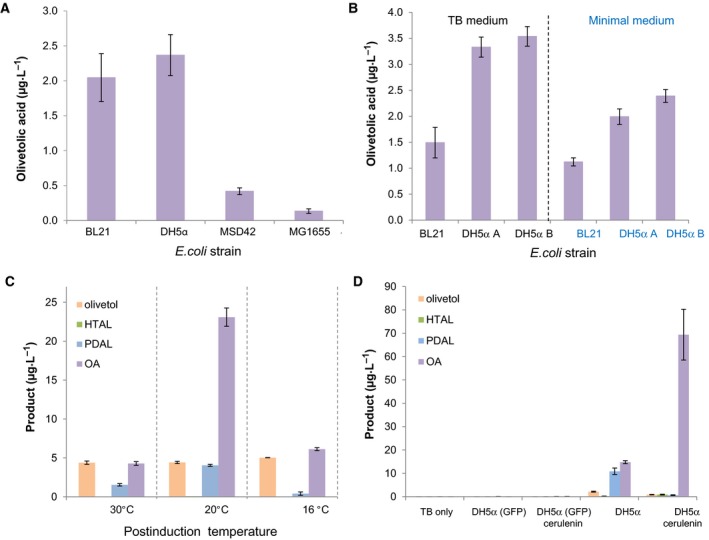
Optimization of *in vivo* olivetolic acid production by varying the (A) *Escherichia coli* strain, (B) culture medium, (C) post induction temperature and (D) the addition of cerulenin. Cultures were incubated in 25 mL TB (except in B where specified) with a starting OD_600 nm_ of 0.1 at 37 °C until the optical density reached 0.8. Recombinant protein expression was induced with anhydrotetracycline (200 nm) ± cerulenin (20 μg·mL^−1^), followed by a further incubation at: 30 °C for (A) and (B), 16–30 °C as specified for (C) and 20 °C for (D) with shaking at 200 r.p.m. Twenty‐four hours after induction, samples were collected and organic solvent soluble products were extracted in ethyl acetate, dried, resuspended in methanol and analysed by LC/MS. Data points for (A) and (B) are the averages of three biological replicates, data points for (C) and (D) are average of two biological replicates, and bars represent standard deviation. TB only: negative control with only culture media; GFP negative control consisting in an *E. coli* strain transformed with a plasmid‐expressing GFP; *E. coli* strains DH5αA and DH5αB were obtained from two different suppliers.

Olivetolic acid production was relatively low under both *in vivo* and *in vitro* conditions. Higher titres have been obtained previously (up to 80 mg·L^−1^) when co‐expressing auxiliary genes involved in up‐regulating precursor supply and optimizing fermentation conditions 9. We performed olivetolic acid production optimization trials targeting the medium composition, host strain, incubation temperature and harvesting time after induction. The best conditions were using *E. coli* strain DH5α as the expression host grown in Terrific broth (TB) media at 20 °C after induction, which successfully increased the olivetolic acid titres 5‐ to 10‐fold (10–20 μg·L^−1^; Fig. 2A–C). A further five‐fold increase in olivetolic acid titres was obtained (70 μg·L^−1^) by the addition of 20 μg·mL^−1^ of cerulenin, a known inhibitor of the fatty acid biosynthesis enzyme, FabF (Fig. 2D) 17. This increases the availability of intracellular malonyl‐CoA, one of the substrates for TKS 17. However, olivetolic acid titres showed a two‐fold decrease 48 h after induction (Fig. 1C). This had been observed previously 9 and olivetolic acid is likely to be degraded by *E. coli.* This was further confirmed by observing a reduction in olivetolic acid loss in TB media alone vs olivetolic acid in the same media containing an *E. coli* DH5α culture (Fig. 1D) 9.

We have demonstrated that our TKS and OAC construct expressed in *E. coli* generate product profiles typical of these enzymes described in earlier studies 7, 8, 9, 18. The production of olivetol instead of olivetolic acid production by TKS alone is consistent with an STS‐like mechanism of cleavage of the C1 thioester linkage between the product and the catalytic triad cysteine (aldol switch) prior to nonenzymatic cyclization. Therefore, further studies into the mode of TKS polyketide intermediate release will be performed in the absence of OAC to eliminate any potential linear polyketide scavenging.

### Structural investigation into olivetol vs olivetolic acid production

**Figure 3:**
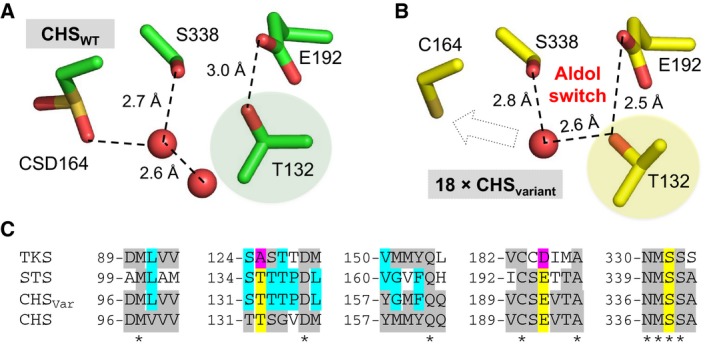
Overview of the aldol switch in type III polyketide synthases. Hydrogen‐bonding network of the active site water proposed to be the catalytic base in the cleavage of the thioester bond between Cys164 and the tetraketide intermediate in (A) wild‐type CHS (PDB: http://www.rcsb.org/pdb/search/structidSearch.do?structureId=1BI5) and (B) variant CHS with STS‐like activity (18 residues altered; PDB: http://www.rcsb.org/pdb/search/structidSearch.do?structureId=1U0W) 12. CSD is a modified cysteine visible in the crystal structure. The dashed arrow in the bottom panel indicates the potential interaction between the catalytic water and Cys164. The shaded residues show the significant differences in orientation of Thr132, the basis of the ‘aldol switch’. (C) Sequence alignment of four type III polyketide synthases within the active site region. Residues highlighted in yellow and magenta are involved in the STS‐like aldol switch, the latter showing differences seen in TKS. CHS_Var_ = variant CHS with STS‐like activity 12.

Our studies have confirmed that TKS is an STS‐like member of the type III polyketide synthases that catalyses C2 → C7 decarboxylative aldol condensation to form olivetol. It cannot generate either olivetolic acid or the equivalent Claisen condensation‐like product. Prior structural and mechanistic studies of CHS and STS identified an aldol switch as being responsible for the differences in the type of cyclization reaction catalysed between highly structurally similar enzymes with the same substrates 12, 15. This involves the presence of a Ser338‐stabilized water molecule, coordinated through a hydrogen‐bonding network with Glu192 and the Thr132 side chain hydroxyl (Fig. 3A,B). This in turn activates it for a thioesterase‐like cleavage of the tetraketide intermediate C1‐Cys164 (CHS numbering) linkage (Scheme 2B). Key to this hydrolysis is the subtle repositioning of the Thr132 side chain hydroxyl, which was caused by a crystallographically determined displacement of a loop of residues 131–137 12, 15. This displacement was seen in the crystal structure of a CHS variant exhibiting STS‐like activity, where 18 amino acids located in loops that differed in conformation between CHS and STS were altered to the equivalent residues found in STS (Fig. 3B) 12. These residues were selected for mutation due to their location in loops with different backbone conformations between CHS and STS.

The potential for water‐mediated cleavage at C1 precyclization is a key factor in determining the type of condensation reaction catalysed. Earlier biomimetic studies found that when C1 was part of a (thio)ester bond, C6 → C1 Claisen cyclization predominates, while C2 → C7 aldol cyclization is favoured when C1 is a free acid 19, 20. Therefore, with STS and TKS, cleavage of the tetraketide intermediate must precede cyclization, in the latter case making it available to interact with OAC to produce olivetolic acid.

A sequence alignment between TKS, STS, CHS wild‐type and an STS‐like CHS variant showed the highly conserved Thr132 and Glu192 residues implicated in the key water hydrogen‐bonding network are substituted for alanine and aspartate in TKS (A125 and D185, respectively; Fig. 3C). Therefore, an STS‐like aldol switch is not present in TKS, due to the substitution of the key hydrogen‐bonding Thr132 for the nonpolar aliphatic residue alanine (Fig. 3C).

This suggests a novel hydrogen‐bonding network for water activation must be present. To investigate this, we determined the co‐crystal structure of TKS in complex with CoA to a high resolution (1.4 Å) to investigate the potential mechanism of active site water activation required to facilitate tetraketide intermediate release from the enzyme. Earlier crystallization and preliminary X‐ray diffraction studies of TKS have been reported, but no crystal structure was solved 21.

**Figure 4:**
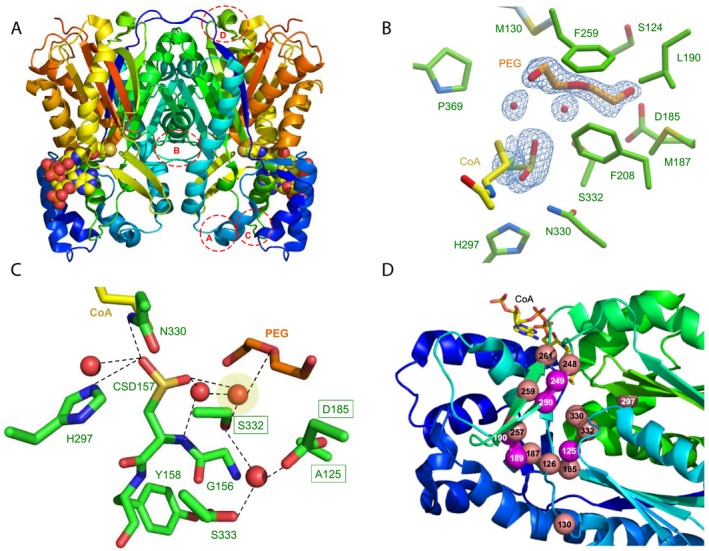
Crystal structure of TKS from *Cannabis sativa*. (A) Overall dimeric structure of TKS shown as a cartoon with CoA as atom coloured spheres. Dashed circles show the regions of deviation from the structure of STS. Regions: A = L65‐Q82; B = S124‐A133; C = R192‐L202 and D = D222‐I230. (B) Active site of TKS, showing density corresponding to oxidized C157, water molecules and polyethylene glycol (PEG). The corresponding omit electron density is shown contoured at 1σ as a blue mesh for the bound PEG ligand, associated waters and the oxidized C157. (C) Hydrogen‐bonding network of active site waters. Residues and ligands are shown as atom coloured sticks with green and orange/yellow carbons, respectively. The catalytic water is highlighted in yellow, and the three equivalent residues in STS important for the aldol switch are boxed. (D) Location of the residues targeted for mutagenesis in TKS. Spheres in salmon were all mutated to alanine (S126, M130, D185, M187, I248, H297, N330, S332, L257, F259 and L261), while magenta residues underwent site‐specific mutagenesis (A125T, C189V, L190T, G249D, G250A). CoA is shown as atom coloured sticks with yellow carbons.

The overall structure adopts a classical thiolase αβαβα five‐layered core (Fig. 4A), and most closely resembles CHS 22 (Dali *Z* score 63.7, rmsd of 0.7 Å over 380 Cα). It contains two dimers in the asymmetric unit, with no significant difference in conformation observed between the four monomers. Only four minor regions in TKS show significant structural deviations from STS (Fig. 4A; loops A–D), highlighting the overall conformational similarity within the type III polyketide synthases. The crystallographic data summary and refinement parameters can be found in Table 1.

**Table 1:** X‐ray crystallographic data collection and refinement statistics. *R*_merge_ = Σ*_hkl_* Σ*_i_*|*I_i_*(*hkl*) − [*I*(*hkl*)]|/Σ*_hkl_* Σ*_i_ I_i_*(*hkl*), where *I_i_*(*hkl*) is the intensity of the *i*th observation of unique reflection *hkl*; Redundancy = total number of reflections/total unique reflections; *R*_work_ = Σ||*F*_obs_| − |*F*_calc_||/Σ|*F*_obs_|, where *F*_obs_ and *F*_calc_ are observed and model structure factors, respectively; *R*_free_ was calculated by using a randomly selected set (5%) of reflections.

Parameters^a^	TKS‐CoA (PDB http://www.rcsb.org/pdb/search/structidSearch.do?structureId=6GW3)
Space group	P 1 2_1_ 1
Cell dimensions	
*a*, *b*, *c* (Å)	71.56, 123.29, 87.94
α, β, γ (^o^)	90, 109.55, 90
Resolution range (Å)	82.87–1.39 (1.43–1.39)
Wavelength (Å)	0.928
Total reflections	938 425 (39 561)
Unique reflections	280 655 (13 838)
Multiplicity	3.3 (2.9)
Completeness (%)	98.40 (96.4)
Mean *I*/σ*I*	13.7 (2.1)
Wilson *B*‐factor (Å^2^)	26.98
*R*_merge_	0.043 (0.420)
*R*_meas_	0.051 (0.521)
CC_1/2_	1.0 (0.8)
*R*_work_	0.118 (0.205)
*R*_free_	0.155 (0.240)
RMS deviations	
Bond angles (°)	1.79
Bond lengths (Å)	0.021
Ramachandran plot	
Allowed region (%)	98.13
Additionally allowed region (%)	1.8
Outliers (%)	0.07
Average *B*‐factor	21.96

a Highest resolution shell is shown in parentheses.

The overlapping initiation/elongation/cyclization cavity is bi‐lobed and contains the conserved Cys157‐His297‐Asn330 catalytic triad. One lobe of this cavity forms the starter molecule‐binding site, while the second accommodates the growing polyketide chain 11. The CoA ligand is positioned with the sulfur moiety in close proximity to the catalytic Cys157 (Fig. 4B). The latter is oxidized to the level of sulfinic acid (CSD) in the crystals, with one of its oxygen atoms occupying the oxyanion hole, mimicking the thioester carbonyl oxygen of bound substrates. Similar modifications have been observed in wild‐type CHS structures (Fig. 3A) 14. The active site region responsible for binding the expanding thioester polyketide moiety is predominantly lined by hydrophobic residues, with the exception of Asp185. Additional electron density was visible within this hydrophobic pocket and was modelled as polyethylene glycol and associated water molecules (Fig. 3B). In view of the multiple conformations of some residues lining the TKS active site, in particular Met187 and Leu257, the volume of the active site is likely to be malleable and able to adapt to the growing polyketide substrate. In the absence of an enzyme–polyketide complex, it is therefore difficult to speculate what structural features underpin product specificity in TKS.

A closer look at the hydrogen‐bonding network in the active site surprisingly shows that it resembles CHS more than STS. The substitution of key conserved STS residues Thr135 and Glu195 for Ala and Asp, respectively, prevents the formation of an aldol switch‐like hydrogen‐bonding network in TKS. In its place, the putative catalytic water molecule (Fig. 4C; shaded) is coordinated to both Ser332, CSD157 and networked through other water molecules in the place of an STS‐like aldol switch. A similar set of interactions is seen in CHS between the catalytic water, CSD164, Ser338 and a second water molecule (Fig. 3A). However, CHS likely does not catalyse tetraketide release prior to cyclization, so the differences in the degree of water activation between TKS, STS and CHS are likely to be subtle and enzyme homologue‐specific. This goes against the idea of a conserved hydrogen‐bonding network ‘switch’ between CHS‐ and STS‐like enzymes as previously suggested 12. Alternatively, it may be differences in the conformation of the tetraketide intermediate that favours one mechanism over the other. No co‐crystal structures are currently available containing the tetraketide (or triketide) intermediates, so it is difficult to make firm conclusions as to the exact discrimination between the mode of water activation in TKS compared to CHS.

### Site‐directed mutagenesis

We performed alanine scanning mutagenesis of 11 residues lining the polyketide binding region of TKS as a complementary approach to investigate key amino acid determinants for catalytic activity (Fig. 4D) 15. This included active site residues S332 and D185 that are structurally implicated in the water activating hydrogen‐bonding network. Soluble protein was obtained for only four of these variants (D185A, M187A, F259A and L261A), and biotransformations were performed in the presence/absence of OAC to see whether the product profiles were altered. All four variants were inactive, suggesting these residues play a more critical role in enzyme stability and/or catalytic activity than initially thought.

**Figure 5:**
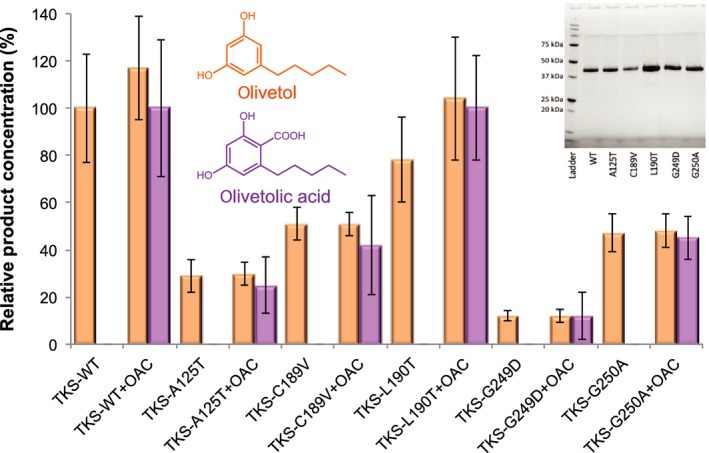
Quantitative LC/MS analysis of wild‐type and variant TKS product profiles from biotransformation reactions with malonyl‐CoA, hexanoyl‐CoA and OAC. Inset: SDS/PAGE analysis of purified wild‐type and variant TKS. Reactions (250 μL) were performed in TKS buffer (25 mm Tris pH 8 containing 150 mm NaCl and 5% glycerol) containing 10 μm hexanoyl‐CoA, 30 μm malonyl‐CoA and 10 μm purified TKS with/without 10 μm OAC for 16 h at 25 °C. The organic soluble intermediates and products of the reactions (200 μL aliquots) were extracted with ethyl acetate (200 μL) and centrifuged at 17 900 ***g*** for 10 min to separate the phases. The organic layer was recovered, and the solvent was removed using a centrifugal evaporator. The products were resuspended in 50% (v/v) methanol (150 μL) and analysed by LC/MS. Reactions also produced variable amounts of PDAL and trace levels of HTAL. Data are expressed as relative product concentration compared to wild‐type enzyme ± OAC data run under the same conditions. The data are averages of technical triplicates of biological duplicates in the case of the A125T, C189V and G249D variants or triplicates in the case of the WT, L190A and G250A variants (2–3 protein purification batches), and error bars represent one standard deviation of the data.

The next approach was to target active site residues that are not highly conserved in the type III polyketide synthases, to see whether these residues are important for discriminating between the cyclization reactions catalysed. We generated four variants of TKS (A125T, C189V, L190T and G249D) where the TKS residue was changed to the equivalent one in CHS (Fig. 3C). Prior studies with CHS showed that altering Gly256 to alanine significantly altered the size of the active site binding product, reducing the yield of cyclized product 11. Therefore, an equivalent variant was generated in TKS (G250A), to see whether there was any impact on olivetol production. In each case, soluble protein was obtained for each variant (Fig. 5 inset), and comparative biotransformations revealed all the enzymes were active and produced primarily olivetol and PDAL (Table S2). Similar to the wild‐type enzyme, only trace levels of HTAL were detected, and near equivalent ratios of olivetol and olivetolic acid were generated in the presence of OAC (Fig. 5). Interestingly, the titres of olivetol were not dramatically impacted by the production of olivetolic acid (OAC‐containing reactions), even though they both utilize the same pool of tetraketide intermediate. No evidence of Claisen cyclization products was detected by LC‐MS, even in the case of variants with CHS substituted residues. Therefore, individual changes to these nonconserved active site residues have not caused a fundamental change in the type of cyclization reaction catalysed.

Surprisingly, the L190T variant had very little impact on olivetol titres, after allowing for batch‐specific variations (Fig. 5). In contrast, when the opposite mutation was performed for CHS (T197L), there was a dramatic reduction in naringenin production from malonyl‐CoA and coumaroyl‐CoA, suggesting this substitution had impacted on its cyclization ability 13. A more dramatic decrease in olivetol (and olivetolic acid) production was detected with the other variants, especially G249D and A125T (8‐ and 3‐fold, respectively). These two small nonpolar residues are located adjacent to the site occupied by PEG in the crystal structure, so the introduction of both steric bulk and charged/polar residues has likely impacted significantly on substrate‐binding. Variants C189V and G250A showed only a ~ 2‐fold reduction in olivetol production, possibly due to the smaller change in steric bulk and the maintenance of a more nonpolar CHS‐like substrate‐binding environment. The ratio of olivetolic acid vs olivetol titres of each variant is similar, suggesting these mutations have directly impacted on the tetraketide intermediate production. This is presumably via altering the binding conformation and/or binding strength of one or both substrates.

Earlier studies demonstrated the plasticity of CHS by the successful switching of the Claisen cyclization reaction to instead possess STS‐like 12 or 2‐PS‐like activity 13 (Scheme 2A) by the substitution of either 18 or 3 amino acids in the enzyme, respectively. In the latter case, the conserved residues were T197L, G256L and S338I (L190, G250 and S332 in TKS, respectively), and this variant no longer accepted its native substrate coumaroyl‐CoA. Instead, it accepted the 2‐PS substrate acetyl‐CoA and produced methylpyrone 13. In the case of TKS single variants, no such alteration in the type of tetraketide condensation reaction was seen. This suggests these residues are either not key players in discriminating between the potential condensation reactions that can be catalysed, or multiple site changes are required to activate the cyclization plasticity seen with other type III polyketide synthases.

## Conclusions

A key challenge in the understanding of the mechanism(s) of action of type III polyketide synthases is identifying the residues responsible for multiple pathway discrimination and the formation of multiple products. This is particularly challenging given the multistep nature of the catalysis and the high sequence and structural identity between homologues displaying alternative cyclization mechanisms to generate distinct chemical scaffolds. The apparent plasticity of TKS with only minor alterations in amino acid sequences demonstrates the subtle nature of the mechanistic discrimination. This involves interplay between multiple residues, an inherently flexible active site designed to accommodate a growing scaffold and easily convertible cyclization mechanism(s) with minimal intervention.

Understanding and predicting the behaviour of individual enzyme homologues is important when designing and optimizing synthetic biology routes towards secondary metabolite production. In the case of microbially sourced therapeutic cannabinoid production, structurally guided mechanistic investigations into multiple cyclization pathway discrimination by polyketide synthases are key to engineering increased yield and purity of target pathway intermediates, thereby impacting the overall titres of tetrahydrocannabinol and CBD. This study has highlighted that the key product discriminating water activating hydrogen‐bonding network or ‘aldol switch’ between CHS and STS/TKS is homologue specific, with no apparent universal STS‐like consensus sequence or mode of catalytic base activation to allow database prediction of decarboxylative aldol cyclization over Claisen condensation. In these types of cases, a combination of classical mechanistic studies coupled with structurally guided mutagenesis of different homologues is required to investigate the cooperative effect of the subtle structural and/or electronic changes required to direct the predominant pathway route(s).

## Materials and methods

### Materials and equipment

Olivetolic acid (2,4‐dihydroxy‐6‐pentylbenzoic acid) standard was purchased from ChemCruz (Santa Cruz biotechnology, Dallas, TX, USA). All other chemicals and solvents were purchased from commercial suppliers and were of analytical grade or better. Media components were obtained from Formedium (Norfolk, UK). Oligonucleotide synthesis and DNA sequencing were performed by Eurofins MWG (Ebersberg, Germany). The strains and plasmid information can be found in Table S3. All oligonucleotide sequences for gene cloning and mutagenesis can be found in Tables S2 and S3, respectively. LC‐MS analysis was performed on an Agilent Technologies (Cheshire, UK) 6560 Ion Mobility Q‐TOF coupled with a 1290 Infinity II UHPLC with a BEH C18 column (130 Å, 1.7 µm, 2.1 mm × 50 mm; Waters, Hertfordshire, UK). Quantitative analysis was carried out on a triple quadrupole tandem mass spectrometer (Xevo TQ‐S; Waters MS Technologies) connected to an Acquity UPLC system with the above BEH C18 column.

### Gene synthesis, sub cloning and mutagenesis

The genes encoding TKS (UniProtKB: http://www.uniprot.org/uniprot/B1Q2B6) and OAC (UniProtKB: http://www.uniprot.org/uniprot/I6WU39) from *C. sativa* were synthesized by GeneArt (Regensburg, Germany), employing codon optimization for increased *E. coli* expression. TKS was subcloned by GeneArt into pETM11, incorporating an N‐terminal TEV cleavable His_6_‐tag. OAC was subcloned into pET42a, which added both a glutathione‐*S*‐transferase (GST) and a His_6_‐tag to the N terminus of the protein. The latter was performed by In‐Fusion cloning (Table S4; Clontech laboratories, Takara Bio, Mountain View, CA, USA) according to the manufacturer’s protocols. A dual expression construct was generated (pBbB2c‐TKS‐OAC) by combining both TKS and OAC and ribosomal binding sites custom designed for *E. coli* into BioBrick vector pBbB2c 16, controlled by a single anhydrotetracycline‐inducible promoter. The individual genes and linearized plasmid backbone (pBbB2c) were amplified by PCR (Table S4) using Phusion Hot Start Flex X2 (New England Biolabs, Ipswich, MA, USA). DNA assembly was performed using the ligase cycling reaction 23.

Alanine scanning mutagenesis was performed for TKS in pETM11 using QuikChange mutagenesis (Novagen, Merck Millipore, Darmstadt, Germany) according to the manufacturer’s protocol (Table S5). The residues targeted were S126, M130, D185, M187, I248, L257, F259, L261, H297, N330 and S332. Additional variants of TKS were generated, namely A125T, C189V, L190T, G249D and G250A. Each mutagenesis was performed using the Q5 site‐directed mutagenesis kit (New England Biolabs) according to the manufacturer’s protocol (Table S5).

In each case, the correct assembly and/or presence of the designed mutations were confirmed by DNA sequencing. The plasmids encoding N‐His_6_‐TKS (wild‐type and variants) and GST‐His_6_‐OAC were transformed into *E. coli* strains BL21(DE3) (New England Biolabs) and ArcticExpress(DE3) (Agilent Technologies), respectively, for protein overexpression. For the dual enzyme plasmid, the *E. coli* NEB5α (New England Biolabs) strain used for *in vivo* studies.

### Protein expression and purification

Both TKS and OAC cultures (6 L) were produced in http://www.rcsb.org/pdb/search/structidSearch.do?structureId=2xYT broth (Formedium) containing kanamycin (40 μg·mL^−1^). Cultures were agitated (200 r.p.m.) at 37 °C until an OD_600 nm_ of 0.6–0.8 was achieved. Protein overexpression was induced by the addition of IPTG (0.1 mm), and cultures were agitated overnight at 16 °C. Cells were harvested by centrifugation (8000 ***g***) for 10 min at 4 °C. The cell pellets (2 mL·g^−1^ pellet) were resuspended in TKS buffer (25 mm Tris pH 8 containing 150 mm NaCl and 5% glycerol) followed by sonication to lyse the cells. Each overexpressed protein was purified using a HisTrap HP column (5 mL; GE Healthcare, Chicago, IL, USA), pre‐equilibrated in TKS buffer. The column was washed stepwise with increasing concentrations of imidazole (10 mL each; 0–50 mm) in TKS buffer. Recombinant protein was eluted with TKS buffer containing 200 mm imidazole (30 mL). Removal of the N‐His_6_‐tag from each enzyme was performed by TEV protease cleavage (1 : 500 w/w) overnight in TKS buffer containing dithiothreitol (1 mm) at 4 °C. Both the TEV protease and cleaved His_6_‐tags were removed by passage through the HisTrap HP column. TKS and OAC eluted in the flow through, while the tag and TEV protease bound to the column. For the TKS variants, purification of each enzyme occurred as above, except for the TEV protease cleavage step. Protein purity was assessed by SDS/PAGE using 12% Mini‐PROTEAN TGX stain‐free precast gels (Bio‐Rad, Hertfordshire, UK). The concentration of each purified enzyme was determined by a Nanodrop (Thermo Fisher, Waltham, MA, USA) or the DC protein assay (Bio‐Rad), according to the manufacturers’ protocols.

### TKS crystallization and structure determination

Co‐crystallization of TKS (10 mg·mL^−1^) with CoA (2 mm) was performed in 0.2 m potassium nitrate containing 20% w/v PEG 3350 for 72 h at 4 °C using the sitting drop vapour diffusion technique. Crystals were cryo‐protected in the mother liquor containing 20% glycerol, followed by flash freezing in liquid nitrogen. The X‐ray data were collected from a single cryo‐protected crystal at Diamond Light Source (Oxford, UK) on beamline I04‐1. The dataset was processed by an automated pipeline implemented in xia2 24, using xds 25 and xscale. Structure determination was performed by molecular replacement in Phaser 26 using CHS (PDB http://www.rcsb.org/pdb/search/structidSearch.do?structureId=1BI5) as the search model. A combination of automated and manual rebuilding in Coot 27 and refinement in Refmac 28 was used to produce each of the refined models. The structures were validated using Molprobity 29 and PDB_REDO 30. The atomic coordinates and structure factors (PDB http://www.rcsb.org/pdb/search/structidSearch.do?structureId=6GW3) have been deposited in the Protein Data Bank, Research Collaboratory for Structural Bioinformatics, Rutgers University, New Brunswick, NJ (http://www.rcsb.org/).

### *In vitro* biotransformations

Reactions (250 μL) were performed in TKS buffer containing 10 μm hexanoyl‐CoA, 30 μm malonyl‐CoA and 10 μm purified wild‐type and variant TKS with/without 10 μm OAC. After an overnight incubation at 25 °C, the organic soluble intermediates and products of the reactions (200 μL aliquots) were extracted with ethyl acetate (200 μL) and centrifuged at 17 900 ***g*** for 10 min to separate the phases. The organic layer was recovered, and the solvent was removed using a centrifugal evaporator (Genevac EZ‐2 plus, Suffolk, UK). The products were resuspended in 50% (v/v) methanol (150 μL), filtered using a PTFE (Supelco 0.2 μm, Sigma Aldrich, Dorset, UK) or PVDF membrane (Millex 0.22 μm, Merck Millipore, Dorset, UK) and analysed by LC/MS. Reactions were performed with technical triplicates of biological replicates (six samples per data point).

### *In vivo* biosynthesis

*In vivo* production of olivetolic acid and by‐products was performed using the *E. coli* strain NEB5α expressing the two‐enzyme construct pBbB2c‐TKS‐OAC. Individual colonies were selected from a freshly transformed culture cultivated overnight on an LB‐chloramphenicol agar plate (25 μg·mL^−1^ chloramphenicol). Starter cultures (5 mL) were grown overnight in TB (tryptone 20 g·L^−1^ and yeast extract 24 g·L^−1^) supplemented with glycerol (4 g·L^−1^) and chloramphenicol (25 μg·mL^−1^) at 37 °C. These cultures were used to inoculate fresh TB medium (25 mL; starting OD_600nm_ of 0.1) and incubated at 37 °C until the OD_600 nm_ reached 0.8. Recombinant protein expression was induced with anhydrotetracycline (200 nm) ± cerulenin (20 μg·mL^−1^), followed by a further incubation at 16–30 °C for 24 h at 200 r.p.m. Cells were harvested periodically (e.g. 4, 24, 48, 72 and 96 h after induction) by centrifugation for 10 min at 4300 ***g***. The intermediates and products were extracted from both the cell and the culture supernatant with an equal volume of ethyl acetate. Organic compounds were dried, resuspended in methanol (200–400 μL; 50%) and analysed by LC/MS as described below. Data points are averages of three biological replicates.

### Mass spectrometry analysis of products

The compounds olivetolic acid, olivetol, PDAL and HTAL were identified from *in vitro* and *in vivo* reactions by LC/MS using a 6560 Ion Mobility Q‐TOF coupled with 1290 Infinity II UHPLC with an Acquity UPLC BEH C18 Column. In this method, the compounds (5 μL) were separated using a mobile phase gradient of solvent A (0.05% formic acid in water) to solvent B (0.05% formic acid in acetonitrile) at a flow rate of 0.6 mL·min^−1^ and a column temperature of 50 °C. In this method, an initial (1–3 min) equilibration in 5% solvent B was followed by a 20‐min gradient to 95% solvent B. Data were acquired in full MS mode, in individual negative acquisitions and scan range of 100–1200 *m*/*z*. Individual compounds were detected at the following *m*/*z* values: HTAL 223.098, PDAL 181.087, olivetolic acid 223.098 and CBGA 359.223 in negative mode, with olivetol 179 in positive mode.

Quantitative analysis was performed on a triple quadrupole tandem mass spectrometer (Xevo TQ‐S; Waters MS Technologies) connected to an Acquity UPLC system (H‐Class; Waters) with compound separation on a BEH C18 column. Compounds were separated using a mobile phase gradient of 70–98% solvent C (0.05% formic acid in methanol) with solvent A, at a flow rate of 0.6 mL·min^−1^ and a column temperature of 45 °C. To separate HTAL, PDAL and olivetolic acid, an initial 70% solvent C was followed by a rapid 0.7‐min gradient to 98% solvent C. To identify olivetol, the initial conditions were 40% C for 3.5 min followed by a gradient to 98% C for 0.5 min. The MS parameters were optimized with a desolvation gas flow of 1000 L·h^−1^, a capillary voltage of 1000 V, desolvation temperature of 600 °C and a source temperature of 150 °C. The MRM transition of 223.01 > 179.12 was used for the quantification of olivetolic acid, 223.07 > 125.09 for HTAL, 181.13 > 97.10 for PDAL, 181.09 > 111.14 for olivetol.

## Conflicts of interest

'The authors declare no conflict of interest.

## Author contributions

LK performed the majority of the cloning and biochemical work. NP performed plasmid construction and tests for the *in vivo* production studies. VK and DL generated the crystal structure. CY provided support for the MS analysis. ET and NSS conceived the initial study design and supervised all aspects of the work. HT collated the data and drafted the manuscript. ET and NSS secured funding and coordinated the programme. All authors approved the final manuscript.

## Supporting information


**Fig. S1.** a) Comparison between the product profiles obtained by TKS and OAC under *in vitro* and *in vivo* conditions obtained via LC‐Q‐TOF MS qualitative analysis.
**Table S1.** Biotransformations of wild type TKS in the presence or absence of OAC.
**Table S2.** Comparative biotransformations of wild type and variant TKS in the presence or absence of OAC.
**Table S3.** Bacterial strains and plasmids.
**Table S4.** Oligonucleotide primer sequences for cloning of tetraketide synthase (TKS) and olivetolic acid cyclase (OAC).
**Table S5.** Oligonucleotide primer sequences for site directed mutagenesis of TKS.
