# Supplementary material for: Structure of the Cannabis sativa olivetol‐producing enzyme reveals cyclization plasticity in type III polyketide synthases
Source: FEBS J. 2019 Oct 28;287(8):1511–24. doi: 10.1111/febs.15089 (PMC7217186; doi:10.1111/febs.15089)
Supplement: Supplementary file 1 — Fig. S1. a) Comparison between the product profiles obtained by TKS and OAC under in vitro and in vivo conditions obtained via LC‐Q‐TOF MS qualitative analysis. Table S1. Biotransformations of wild type TKS in the presence or absence of OAC. Table S2. Comparative biotransformations of wild type and variant TKS in the presence or absence of OAC. Table S3. Bacterial strains and plasmids. Table S4. Oligonucleotide primer sequences for cloning of tetraketide synthase (TKS) and olivetolic acid cyclase (OAC). Table S5. Oligonucleotide primer sequences for site directed mutagenesis of TKS. [file FEBS-287-1511-s001.zip › febs15089-sup-0001-Supinfo.pdf]

FEBS PRESS
science publishing by scientists
WILEY
The FEBS Journal
www.febsjournal.org

Structure of the *Cannabis sativa* olivetol-producing enzyme reveals cyclization plasticity in type III polyketide synthases

Lewis J. Kearsey, Nicole Prandi, Vijaykumar Karuppiah, Cunyu Yan, David Leys, Helen Toogood, Eriko Takano and Nigel S. Scrutton

DOI: 10.1111/febs.15089

Supporting Information

Structure of the *Cannabis sativa* olivetol-producing enzyme reveals cyclization plasticity in Type III polyketide synthases

Lewis J. Kearsey,[1] Nicole Prandi,[1] Vijaykumar Karuppiah,[1†] Cunyu Yan,[2] David Leys,[1] Helen Toogood,[1] Eriko Takano[1,2,3] and Nigel S. Scrutton[1,2,3*]

[1]Manchester Institute of Biotechnology and School of Chemistry, [2]BBSRC/EPSRC Synthetic Biology Research Centre SYNBIOCHEM, and [3]EPSRC/BBSRC Future Biomanufacturing Research Hub, The University of Manchester, Manchester, M1 7DN, UK.

[†]Present address: Immunocore Limited, Abingdon, Oxfordshire, OX14 4RY, UK

*Corresponding author

Supporting Figures

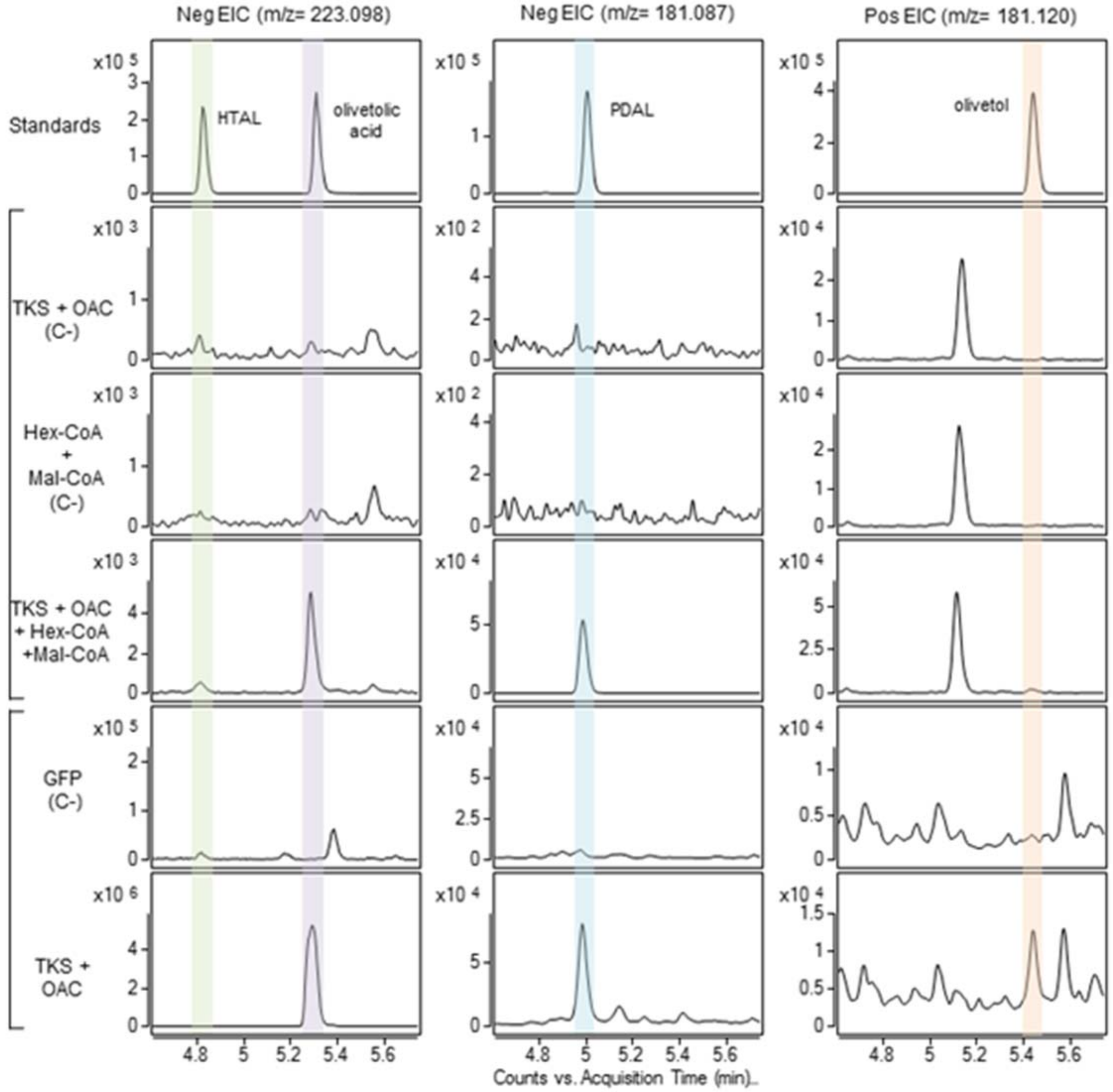

Figure S1. a) Comparison between the product profiles obtained by TKS and OAC under *in vitro* and *in vivo* conditions obtained via LC-Q-TOF MS qualitative analysis. Standards: commercial chemical standards. *In vitro:* products obtained in a biotransformation assay with purified TKS in the presence or absence of OAC. *In vivo*: products obtained when expressing the TKS and OAC in *E. coli,* grown at 20° C after induction in TB media. TKS +OAC: Extract from a culture transformed with a vector expressing TKS and OAC. GFP: extracts from a culture transformed with the vector expressing GFP as a negative control. Neg EIC: Samples run in ESI negative ion mode, (m/z= 223.098 and 181.087) Pos EIC: Samples run in ESI positive ion mode, trace shown is extracted ion chromatogram (m/z=181.12). The retention times of authentic standards are HTAL (4.8 min), PDAL (5.03 min), OLA (5.3 min) and olivetol (5.59 min).

Supporting Tables

Table S1. Biotransformations of wild type TKS in the presence or absence of OAC.

Enzyme(s)	Product (nM)			
	**Olivetol**	**Olivetolic acid**	**PDAL[1]**	**HTAL**
TKS_{WT}	416 ± 96	ND	(270 ± 152)<br>(834 ± 138)	5.4 ± 3.7
TKS_{WT} + OAC	487 ± 92	13.7 ± 4.0	(225 ± 101)<br>(171 ± 121)	4.1 ± 4.0

Reactions (250 µL) were performed in TKS buffer (25 mM Tris pH 8 containing 150 mM NaCl and 5% glycerol) containing 10 µM hexanoyl-CoA, 30 µM malonyl-CoA and 10 µM purified TKS with/without 10 µM OAC for 16 h at 25 °C. The organic soluble intermediates and products of the reactions (200 µL aliquots) were extracted with ethyl acetate (200 µL), and the solvent was removed using a centrifugal evaporator. The products were resuspended in 50% methanol (150 µL) and analysed by LC/MS. [1]Data in parentheses are triplicates of only one batch of purified protein. The remaining data are averages of triplicates of biological duplicates or triplicates. ND = none detected.

Table S2. Comparative biotransformations of wild type and variant TKS in the presence or absence of OAC.

Enzyme(s)	Relative product concentration (%)	
	**Olivetol**	**Olivetolic acid**
TKS_{WT}	100 ± 23	ND
TKS_{WT} + OAC	117 ± 22	100 ± 29
TKS_{A125T}	29 ± 7	ND
TKS_{A125T} + OAC	30 ± 5	25 ± 12
TKS_{C189V}	51 ± 7	ND
TKS_{C189V} + OAC	51 ± 5	42 ± 21
TKS_{L190T}	78 ± 18	ND
TKS_{L190T} + OAC	104 ± 26	100 ± 22
TKS_{G249D}	12 ± 2	ND
TKS_{G249D} + OAC	12 ± 3	12 ± 10
TKS_{G250A}	47 ± 8	ND
TKS_{G250A} + OAC	48 ± 7	45 ± 9

Reactions (250 µL) were performed in TKS buffer (25 mM Tris pH 8 containing 150 mM NaCl and 5% glycerol) containing 10 µM hexanoyl-CoA, 30 µM malonyl-CoA and 10 µM purified TKS with/without 10 µM OAC for 16 h at 25 °C. The organic soluble intermediates and products of the reactions (200 µL aliquots) were extracted with ethyl acetate (200 µL), and the solvent was removed using a centrifugal evaporator. The products were resuspended in 50% methanol (150 µL) and analysed by LC/MS. Reactions also produced variable amounts of PDAL and trace levels of HTAL. [1]Data in parentheses are triplicates of only one batch of purified protein. The remaining data are averages of triplicates of biological duplicates or triplicates (2-3 protein purification batches). ND = none detected. Data are expressed as relative product concentration compared to wild-type enzyme +/- OAC data run under the same conditions.

Table S3. Bacterial strains and plasmids.

Strain or plasmid	Description	Source or Reference
***E. coli* strain**		
NEB5α	fhuA2 Δ(*argF-lacZ*)U169 *phoA* glnV44 Φ80 Δ(*lacZ*)M15 gyrA96 *recA1* relA1 *endA1* thi-1 hsdR17	New England Biolabs
BL21(DE3)	fhuA2 [lon] *ompT* gal (λ DE3) [*dcm*] Δ*hsdS*; λ DE3 = λ sBamHIo ΔEcoRI-B int::(*lacI*::PlacUV5::T7 gene1) i21 Δnin5	New England Biolabs
ArcticExpress(DE3)	B F^- *ompT hsdS*(r^- m^-) *dcm*$^+$ Tet^r *gal* λ(DE3) *endA* Hte [*cpn10* BB *cpn60* Gentr]	Agilent Technologies
MSD42	(MG1655) fhuACDB$^-$, endA$^-$, ΔrecA	[1]
MG1655	F- lambda- *ilvG- rfb*-50 *rph*-1 Serotype: OR:H48:K-	[2]
**Plasmids**		
TKS_pETM11	N-His$_6$-TKS wild-type; TEV protease cleavable; T7; Kan^R	This study
TKS$_{S126A}$_pETM11	N-His$_6$-TKS variant S126A; TEV protease cleavable; T7; Kan^R	This study
TKS$_{M130A}$_pETM11	N-His$_6$-TKS variant M130A; TEV protease cleavable; T7; Kan^R	This study
TKS$_{D185A}$_pETM11	N-His$_6$-TKS variant D185A; TEV protease cleavable; T7; Kan^R	This study
TKS$_{M187A}$_pETM11	N-His$_6$-TKS variant M187A; TEV protease cleavable; T7; Kan^R	This study
TKS$_{I248A}$_pETM11	N-His$_6$-TKS variant I248A; TEV protease cleavable; T7; Kan^R	This study
TKS$_{L257A}$_pETM11	N-His$_6$-TKS variant L257A; TEV protease cleavable; T7; Kan^R	This study
TKS$_{F259A}$_pETM11	N-His$_6$-TKS variant F259A; TEV protease cleavable; T7; Kan^R	This study
TKS$_{L261A}$_pETM11	N-His$_6$-TKS variant L261A; TEV protease cleavable; T7; Kan^R	This study
TKS$_{H297A}$_pETM11	N-His$_6$-TKS variant H297A; TEV protease cleavable; T7; Kan^R	This study
TKS$_{N330A}$_pETM11	N-His$_6$-TKS variant N330A; TEV protease cleavable; T7; Kan^R	This study
TKS$_{S332A}$_pETM11	N-His$_6$-TKS variant S332A; TEV protease cleavable; T7; Kan^R	This study
TKS$_{A125T}$_pETM11	N-His$_6$-TKS variant A125T; TEV protease cleavable; T7; Kan^R	This study
TKS$_{C189V}$_pETM11	N-His$_6$-TKS variant C189V; TEV protease cleavable; T7; Kan^R	This study
TKS$_{L190T}$_pETM11	N-His$_6$-TKS variant L190T; TEV protease cleavable; T7; Kan^R	This study
TKS$_{G249D}$_pETM11	N-His$_6$-TKS variant G249D; TEV protease cleavable; T7; Kan^R	This study
TKS$_{G250A}$_pETM11	N-His$_6$-TKS variant G250A; TEV protease cleavable; T7; Kan^R	This study
OAC_pET42a	N-GST-His$_6$-OAC wild-type; T7; Kan^R	This study
TKS-OAC-pBbB2c	TKS and OAC; *tet* promoter; Chl^R	This study
Kan^R = kanamycin resistant; Chl^R = chloramphenicol resistant.		

Table S4. Oligonucleotide primer sequences for cloning of tetraketide synthase (TKS) and olivetolic acid cyclase (OAC).

Primer name	Template	Modification	5’ Sequence
Sub cloning of OAC into pET42a			
Forward	OAC	None	CATCACCATCACTCCATGAGCGATTACGACATCC
Reverse	OAC	None	GGTGGTGGTGCTCGATTATTTACGCGGGGTATAGTC
Construction of pBbB2c-TKS-OAC			
pMA-TB1Q2B6F	TKS	5' Phos	CCTTAGGTCAATCCACAAAAGCG
pMA-TB1Q2B6R	TKS	5' Phos	TCAATATTTTATAGGAACCGATCGTACCAC
pTwistI6WU39F	OAC	5' Phos	TCGAACTTTCGTAATCGTAAAGTCGC
pTwistI6WU39R	OAC	5' Phos	TTATTTACGCGGGGTATAGTCAAAAATCAG
pBbopenF	Vector	5' Phos	GGATCCAAACTCGAGTAAGG
pBbopen-R	Vector	5' Phos	CTTCTTAAAAGATCTTTTGAATTC

Table S5. Oligonucleotide primer sequences for site directed mutagenesis of TKS.

Mutation	Direction	5' Sequence
Alanine scanning mutagenesis		
S126A	Forward	CACTTAATTTTTACCTCAGCGGCGACTACCGATATGCCTGGTG
	Reverse	CACCAGGCATATCGGTAGTCGCCGCTGAGGTAAAAATTAAGTG
M130A	Forward	CCTCAGCGTCGACTACCGATGCGCCTGGTGCCGACTATCATTG
	Reverse	CAATGATAGTCGGCACCAGGCGCATCGGTAGTCGACGCTGAGG
D185A	Forward	GTGTTCTGGCCGTTTGCTGTGCTATCATGGCATGCCTGTTTCG
	Reverse	CGAAACAGGCATGCCATGATAGCACAGCAAACGGCCAGAACAC
M187A	Forward	GCCGTTTGCTGTGATATCGCGGCATGCCTGTTTCGTGG
	Reverse	CCACGAAACAGGCATGCCGCGATATCACAGCAAACGGC
I248A	Forward	CAAATAGCGAAGGCACTGCCGGGGGCCACATCCGCG
	Reverse	CGCGGATGTGGCCCCCGGCAGTGCCTTCGCTATTTG
H297A	Forward	GCCACATCCGCGAAGCTGGAGCGATTTTTGACCTGCATAAAG
	Reverse	CTTTATGCAGGTCAAAAATCGCTCCAGCTTCGCGGATGTGGC
N330A	Forward	CCGCGAAGCTGGACTGATTGCTGACCTGCATAAAGATGTC
	Reverse	GACATCTTTATGCAGGTCAGCAATCAGTCCAGCTTCGCGG
S332A	Forward	GCTGGACTGATTTTTGACGCGCATAAAGATGTCCCGATG
	Reverse	CATCGGGACATCTTTATGCGCGTCAAAAATCAGTCCAGC
L257A	Forward	CAGCATTTTCTGGATAACGGCTCCGGGCGGCAAAGCTATC
	Reverse	GATAGCTTTGCCGCCCGGAGCCGTTATCCAGAAAATGCTG
F259A	Forward	CGTGTTGAGCGAACATGGTGCTATGAGCTCCTCAACAGTC
	Reverse	GACTGTTGAGGAGCTCATAGCACCATGTTCGCTCAACACG
L261A	Forward	GAGCGAACATGGTAATATGGCCTCCTCAACAGTCCTATTC
	Reverse	GAATAGGACTGTTGAGGAGGCCATATTACCATGTTCGCTC
Site directed mutagenesis at other sites		
A125T[1]	Forward	GGTAGTCGAggtTGAGGTAAAAATTAAGTGTG
	Reverse	GATATGCCTGGTGCCGAC
C189V[1]	Forward	CACGAAACAGcacTGCCATGATATCACAGC
	Reverse	GTCCTTCTGAGAGCGACTTAG
L190T[1]	Forward	GACCACGAAAggtGCATGCCATGATATC
	Reverse	CTTCTGAGAGCGACTTAGAAC
G249D[1]	Forward	GGATGTGGCCatcGATAGTGCCTTC
	Reverse	GCGAAGCTGGACTGATTTTTG
G250A[1]	Forward	CGGATGTGcgcCCCGATAGTG
	Reverse	CGAAGCTGGACTGATTTTTGACC

[1]Lower case letters indicate the mutated codon.

References

1. Kolisnychenko V, Plunkett G, Herring CD, Feher T, Posfai J, Blattner FR & Posfai G (2002) Engineering a reduced *Escherichia coli* genome., *Genome Res* 12, 640–647.
2. Soupene E, van Heeswijk WC, Plumbridge J, Stewart V, Bertenthal D, Lee H, Prasad G, Paliy O, Charernnoppakul P & Kustu S (2003) Physiological studies of *Escherichia coli* strain MG1655: Growth defects and apparent cross-regulation of gene expression, *J Bacteriol* 185, 5611-5626.
